# P369: Financial benefits after the implementation of antimicrobial copper in intensive care units (ICUs)

**DOI:** 10.1186/2047-2994-2-S1-P369

**Published:** 2013-06-20

**Authors:** P Efstathiou, E Kouskouni, S Papanikolaou, K Karageorgou, Z Manolidou, M Tseroni, E Logothetis, C Petropoulou, V Karyoti

**Affiliations:** 1 National Health Operations Centre, Athens, Greece; 2 Medical School of the University of Athens, Microbiology laboratory of Aretaieio Hospital, Ministry of Health, Athens, Greece; 3 ICU, Peiraikon General Hospital, Athens, Greece; 4 “Agia Sofia” Childrens Hospital (NICU), Ministry of Health, Athens, Greece

## Objectives

Aim of this study was to evaluate the reduction on Intensive Care Unit (ICU) microbial flora after the antimicrobial copper alloy (Cu^+^) implementation as well as the effect on financial - epidemiological operation parameters.

## Methods

Medical, epidemiological and financial data into two time periods, before and after the implementation of copper (Cu 63% - Zn 37%, Low Lead) were recorded and analyzed in a General ICU. The evaluated parameters were: the importance of patients' admission (Acute Physiology and Chronic Health Evaluation - APACHE II and Simplified Acute Physiology Score - SAPS), microbial flora's record in the ICU before and after the implementation of Cu^+^ as well as the impact on epidemiological and ICU's operation financial parameters.

## Results

During December 2010 and March 2011 and respectively during December 2011 and March 2012 comparative results showed statistically significant reduction on the microbial flora (CFU / ml) by 95% and the use of antimicrobial medicine (per day per patient) by 30% (p = 0,014 ) as well as patients hospitalization time and cost.

## Conclusion

The innovative implementation of antimicrobial copper in ICUs contributed to their microbial flora significant reduction and antimicrobial drugs use reduction with the apparent positive effect (decrease) in both patients hospitalization time and cost. Under the present circumstances of economic crisis, survey results are of highest importance and value.

## Disclosure of interest

None declared

